# The Effects of Exercise on IL-6 Levels and Cognitive Performance in Patients with Schizophrenia

**DOI:** 10.3390/diseases7010011

**Published:** 2019-01-22

**Authors:** Pablo Gómez-Rubio, Isabel Trapero

**Affiliations:** Departamento de Enfermeria, Facultad de Enfermería y Podología, Univerdidad de Valencia, 46010 Valencia, Spain; Isabel.Trapero@uv.es

**Keywords:** interleukin-6, schizophrenia, physical exercise, immune system

## Abstract

Exercise plays an important role in brain plasticity, leading to improvements in cognitive function and delaying the cognitive deterioration of healthy people. These effects can be observed in individuals with schizophrenia through improvements in their performance in cognitive tasks and a decrease in the symptomology of the disease. In this review we examine the current evidence for the roles that exercise and the immune system play in patients with schizophrenia, and specifically analyze the interleukin-6 (IL-6) pathway as a potential mechanism resulting in these positive effects. Inflammation and high levels of IL-6 are associated with both the severity of schizophrenia and the cognitive impairment suffered throughout the disease. Performing regular exercise can modulate IL-6 by lowering its basal levels and by causing lower acute increases in the plasma levels of this cytokine in response to exercise (an anti-inflammatory response to physical exertion). Although there is evidence for the positive effects of physical exercise on schizophrenia, more studies will be required to better understand how variation in different exercise parameters affects both the acute and chronic plasma levels of IL-6.

## 1. Introduction

Schizophrenia affects approximately 24 million people around the world and is considered a severe psychiatric disorder characterized by ‘positive symptoms’ (hallucinations and delusions), ‘negative symptoms’ (including avolition and anhedonia), and cognitive deficits (such as deficiencies in perception, memory, and attention) [1]. Despite extensive research on this disorder, standard treatments including medication and cognitive behavioral therapy have not been shown to be effective, mainly in relation to negative symptoms and cognitive deficits [2].

One of the reasons why current treatments are ineffective is that schizophrenia is still sometimes misunderstood because of the complexity of its pathogenesis [3]. The manifold interactions between the immune system and the nervous system could underlie the development of schizophrenia as part of a complex mechanism. Evidence suggests that an increasing level of a stressing hormone may activate the inflammatory arm of the immune system and, hence, trigger the expression of genes responsible to elicit a chronic and low-grade inflammation state [4]. The interest in immune/inflammatory changes and their associated oxidative consequences as a potential determinant/key in the pathophysiology of schizophrenia has been recently restored. The latest evidence supports a model which describes that the onset of oxidative stress stimuli and a consequent immune dysfunction may alter cellular homeostasis which, in turn, may determine an aberrant growth of interneurons and, consequently, a psychotic symptomatology [5]. Within this context, the possible relationship between a psychotic breakdown evolving in schizophrenia and inflammation proteins, such C-reactive protein (CRP), has been investigated [6]. CRP is thought to assist in complement binding to foreign and damaged cells and to affect the humoral response to disease [7]. It is also believed to play an important role in innate immunity, as an early defence system against infections [8], but future research is needed to investigate the relationships between CRP levels and cytokines [9]. The current evidence, supported by genetic studies, relates this disorder to an imbalance in cytokines and, consequently, to dysregulated triggering of inflammatory processes, with IL-6 being at the center of many of these studies [10].

This new evidence regarding the possible relationships between the immune system and schizophrenia, together with the lack of efficacy of standard treatments, means that exercise is now being considered as a possible treatment for this disease. This is because, when combined with the adequate prescription of other treatments, it has beneficial effects on the immune system. In fact, some studies have already been published that show a reduction in the symptomology of patients with schizophrenia thanks to exercise interventions [11].

In this article we examine the relationships between schizophrenia, exercise, and the immune system, and specifically look at the current evidence regarding IL-6 as one of the potential mechanisms through which exercise could produce improvements in the symptoms of schizophrenia.

Our search strategy conducted an electronic database search of CINAHL, Scopus, Web of Science, MEDLINE, Embase, the Cochrane Library, and PubMed from inception on 10 May 2018. The keyword search terms used were as follows: ‘schizophrenia’ and ‘exercise’ or ‘physical activity’ and ‘cognitive’ or ‘cognition’ and ‘IL-6′. As we did not find enough information, we carried out two more searches. First of all we used the following keyword search terms: ‘schizophrenia’ and ‘exercise’ or ‘physical activity’ and ‘IL-6′. Secondly, we used the following keyword search terms: ‘schizophrenia’ and ‘cognitive’ or ‘cognition’ and ‘IL-6′. Google Scholar and the reference lists of retrieved articles were also searched in order to identify any additional relevant publications.

## 2. Schizophrenia and Exercise

### 2.1. Exercise in Relation to Cognition

**Figure 1:**
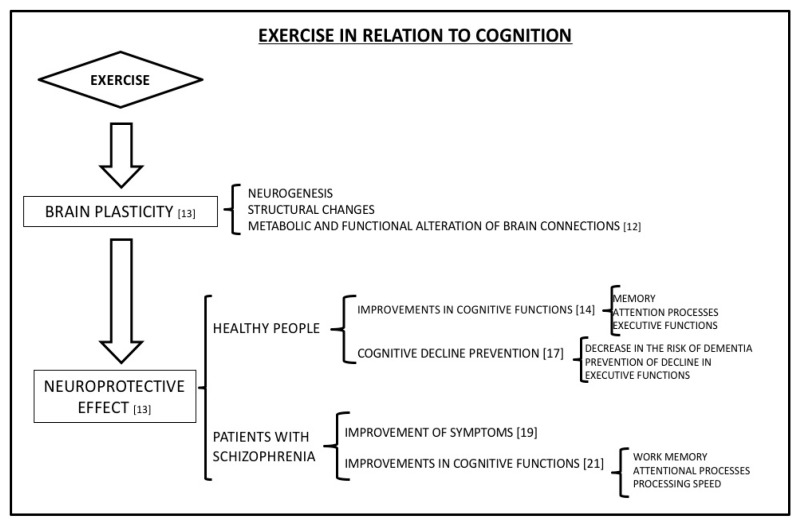
Exercise has a potential role in brain plasticity that leads to a neuroprotective effect in healthy people and in patients with schizophrenia. This neuroprotective effect has positive consequences in the improvement of cognitive functions, in the delay of cognitive deterioration, and in the improvement of the symptomatology of schizophrenia.

There is already a lot of evidence to suggest that exercise affects brain plasticity by inducing neurogenesis and causing structural changes [12]. Exercise plays an important role in the metabolic, structural, and functional alteration of brain connections, and is an important factor in brain plasticity that can be protective against the cognitive decline consequent to aging [13]. These effects are reflected in cognitive functioning at the cerebral level, both in the improvement of different cognitive functions and in the prevention of the cognitive deterioration that occurs because of aging (Figure 1).

Improvements in cognitive functions have mainly been observed in terms of memory (implicit memory tasks, immediate memory, and recognition), attention processes, and in executive functions [14]. Better performance in verbal, perceptual, and arithmetic tasks has also been shown in children who regularly perform exercise compared with sedentary children [15].

As already mentioned, numerous studies have shown that exercise prevents the cognitive decline related to age [16], reduces the risk of developing dementia, and slows the decline in executive-function impairment [17]. The neuroprotective effect induced by exercise has also been observed in people with dementia and cognitive disability [18], reducing their symptoms and improving cognitive functions in both young people and adults [19].

### 2.2. Schizophrenia and Exercise

In relation to schizophrenia, current studies indicate that exercise can reduce psychiatric symptoms and improve cognitive functions in different subdomains, as reported in the European Psychiatric Association (EPA) guidance [20]. The benefits of exercise for treating this disorder are related to its effects at the level of the brain, and the protective effect produced in the hippocampus by exercise are similar both in patients with schizophrenia and in healthy adults [11] (Figure 1). Several different studies have observed that these changes caused by exercise at the level of the brain improve both the positive and negative symptoms of schizophrenia. The reduction in the incidence of negative symptoms is especially relevant because standard medication is less effective at treating them [19].

Furthermore, there is evidence that exercise also improves performance in tasks involving working memory, processing speed and attentional processes [21]. Specifically, undertaking light physical activity positively correlated with cognitive performance, while moderate and vigorous physical activity was associated with an improvement in the symptoms of schizophrenia [22]. The exact mechanism or mechanisms that underlie this exercise-mediated cognitive improvement are currently unknown, but several studies have indicated that the immune system as is likely involved.

## 3. Schizophrenia, Exercise and the Immune System

### 3.1. The Immune System in Relation to Cognition

Several studies have found that the immune system is involved in the homeostatic modulation of neurogenesis [23], observing that, among other mechanisms, stimulation of peripheral-level cytokines alters several key neurotransmitters that optimize neurogenesis [24]. The positive effect that the immune system has in this context is conditioned by complex interactions between the brain cells that perform immune functions and neurons via neurotransmitters and pro- and anti-inflammatory cytokines [25].

Thus, these improvements resulted in gains in learning and memory processes [26] and produced an inverse association between inflammatory processes and performance in spatial reasoning, short-term memory, verbal learning, and executive-function tests [27]. Many studies point out that an adequate balance between pro- and anti-inflammatory cytokines underlies these improvements in cognitive processes by modulating and consolidating the reorganization of neural networks [25].

Nevertheless, it has also been shown that cytokine levels and the balance between pro- and anti-inflammatory cytokines vary over an individual’s lifetime [28], suggesting that production of high amounts of inflammatory cytokines and a decrease in anti-inflammatory levels increase the central inflammatory response, damaging synaptic plasticity [13] and consequently also augmenting age-associated worsening of cognitive performance. Thus, certain inflammatory biomarkers are already used to predict cognitive decline [28].

### 3.2. The Immune System and Schizophrenia

Inflammation is currently considered a potential mechanism in the development and progression of schizophrenia [29]. Patients with schizophrenia have increased levels of inflammatory cytokine markers and, more specifically, this increase may be related to the negative symptoms of the disease [30]. Therefore, an inadequate balance between pro- and anti-inflammatory cytokines would cause immune system dysfunction, impairing neurodevelopment and deregulating brain neurotransmission in patients with schizophrenia [10].

### 3.3. The Immune System and Exercise

Maintaining a good balance between pro- and anti-inflammatory cytokines could be one of the mechanisms through which exercise exerts its immunoprotective and immunoregulatory effects [31]. Exercise, as a stressor, induces different immune responses associated with the production of interleukins, thus eliciting an anti-inflammatory response through inflammation [32]. There is ample evidence that exercise is associated with a systemic reduction in inflammation [14].

Additionally, new genetic studies suggest that exercise may activate genes involved in the production of leukocytes as well as those that downregulate inflammation. Although this mechanism is not fully understood, studies are beginning to appear that indicate that exercise parameters such as intensity and duration are important factors in sufficiently regulating cytokines to induce this protective anti-inflammatory response both in healthy individuals and in those with different pathologies [33]. However, the interaction of multiple factors in the immune system mean that doubts remain about the exact mechanisms which interrelate it with schizophrenia and exercise, although recent work has emerged highlighting an important role for IL-6 in this context. 

## 4. Schizophrenia, Exercise and IL-6

### 4.1. IL-6 and Cognition

**Figure 2:**
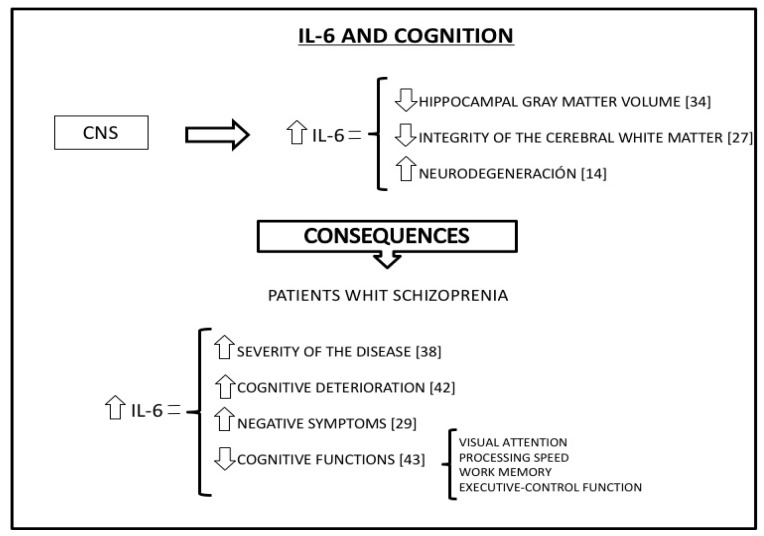
IL-6 has numerous effects in the central nervous system (CNS). These effects have different consequences in the patients with schizophrenia.

Numerous studies have published evidence regarding the role of IL-6 in the inflammatory processes which interconnect the immune system with the central nervous system (CNS). IL-6 levels inversely correlate with hippocampal gray matter volume [34] as well as with the integrity of the white matter in the brain [27]. One of the mechanisms by which the immune system influences cognitive function performance, neurodegeneration, and cognitive functionality, involves interactions between IL-6 and the CNS (Figure 2).

Regarding cognitive function, there is evidence that high levels of peripheral IL-6 negatively affects memory and learning [34], is associated with generally poor cognitive performance [35], and that cognitive-task performance is lower in acute inflammatory processes involving increased levels of IL-6 [36]. It is also important that the peripheral inflammatory mediators such as IL-6 can cross the blood–brain barrier and modulate central inflammatory processes, leading to increased neurodegeneration and negatively influencing cognitive function [27]. Specifically, the presence of high basal IL-6 levels increases the risk of future cognitive age-related decline [37,38]. Increased IL-6 has also been observed in dementia and other diseases characterized by cognitive decline, as well as in depressive disorders [39]. Thus, presence of low levels of IL-6 may reduce the risk of developing neurodegenerative diseases [14]. 

However, even though most studies discuss the negative effects of elevated IL-6 levels, there is evidence that moderate levels of IL-6 may also have an anti-inflammatory and protective effect [40].

### 4.2. Il-6 and Schizophrenia

There is a lot of evidence in the literature regarding immune system deregulation and the presence of high levels of pro-inflammatory cytokines such as IL-6 in patients with schizophrenia [41]. Moreover, high levels of IL-6 are associated with the altered brain morphology and the severity of the disease [38] as well as with the continuing cognitive deterioration [42] and decline in visual attention, processing speed, working memory, and executive-control function [43] seen as schizophrenia progresses (Figure 2). Looking more specifically at the different symptoms of schizophrenia, it appears that high levels of inflammation and IL-6 are associated with the disease’s negative symptoms [29].

### 4.3. Il-6 and Exercise

Exercise induces an increase in the plasma levels of different cytokines, including IL-6 [44]. In the year 2000, Steenberg et al. published the first study to show that the skeletal-muscle activity connected with exercise increases plasmatic IL-6 levels [45]. Following studies showed that the magnitude of this muscle-fiber derived plasma IL-6 elevation caused by exercise correlates with different parameters such as the intensity and duration of the physical activity [46].

In addition to the acute effect that exercise has on the increase of plasma IL-6 levels, different studies have also shown that over time, following a protocol of prolonged exercise, causes baseline IL-6 levels to decrease [41]. This reduction is also related to the intensity and duration of physical exercise as well as other variables such as the fitness levels of the study participants [47]. People who perform regular exercise in their daily lives show lower baseline IL-6 levels compared to those who are sedentary [45].

Although, as we have already mentioned, most studies associate IL-6 with proinflammatory responses, numerous studies have also observed that the acute increase in IL-6 levels caused by exercise gives rise to an anti-inflammatory response by inhibiting, for example, the activation of tumor necrosis factor alpha (TNFα) whose activity is highly proinflammatory [40] and by stimulating the production of other predominantly anti-inflammatory immune system molecules [48].

## 5. Discussion

The evidence available in the scientific literature strongly corroborates that exercise has numerous beneficial effects on the CNS; it exerts a neuroprotective effect, delays and slows the decline in cognitive performance produced by age, and improves and optimizes different cognitive functions such as learning, memory, and executive functions [12]. As set out by the EPA guide [20], the benefits related to exercise seen in healthy people are also observed in patients with schizophrenia, both in terms of symptomatology and in improvements in cognitive function.

Nonetheless, many unresolved questions remain about the specific mechanisms through which exercise mediates these beneficial effects on the cognitive system, both generally in healthy people [29] and especially, in patients with schizophrenia [49]. However, the evidence suggesting that the immune system plays a predominant role in these effects is mounting. Numerous studies have revealed that exercise has an anti-inflammatory effect that positively affects neuroplasticity [13]. Related to exercise, several studies have revealed that pro- and anti-inflammatory cytokines must be properly balanced so that the immune system can exert positive or negative effects on brain neuroplasticity, to modulate and consolidate the reorganization of neural networks, and the benefits that exercise has on schizophrenia are because it mediates cerebral structural changes via the immune system [25].

When pro- and anti-inflammatory cytokines are adequately balanced, the cytokine IL-6 appears to play a significant role in both pro- and anti-inflammatory responses, depending on the levels of its expression [40]. Moreover, IL-6 can cross the blood–brain barrier where it can modulate central inflammatory processes and, if its levels are too high, it results in increased neurodegeneration and negatively influences cognitive function [27]. Furthermore, recently-published reviews indicate that high levels of inflammation and IL-6 are related to the negative symptoms of schizophrenia [29].

We now know that the activity generated in the skeletal muscles by exercise is one of the main mechanisms by which the plasma and baseline levels of IL-6 can be modulated [44]. Although there are doubts about the exact mechanism through which this modulation occurs, several studies have shown that as a consequence of exercise there is an acute increase in the plasma levels of IL-6 [40]. This increase occurs as a result of the muscle damage caused by exercise and depends on the different parameters of the exercise performed, including its intensity, duration, and the types of muscle contraction involved, with higher levels of IL-6 resulting from exercise which causes homeostasis to destabilize [50].

Conversely, regularly performing exercise reduces the peak of these increases in IL-6 levels, thus attenuating this acute effect. This means that the final increases in plasma IL-6 levels are a function of the fitness status of each person and their individual level of adaptation to exercise [50]. This decreased IL-6 plasma level peak, seen after only one exercise session in individuals who regularly do exercise, is also observed in the basal levels of IL-6, so that people who regularly exercise have lower baseline levels than those who are sedentary [44].

Therefore, the acute response to a single dose of exercise leads to an increase in plasma levels of IL-6, but the chronic response to regular exercise produces both a minor increase in this acute response and a decrease of the basal levels of IL-6. Regarding the effects of increased levels of IL-6, although most studies refer to its negative effects [39] and its pro-inflammatory properties, several studies also suggest that the moderate increases in IL-6 levels provoked by exercise could lead to an anti-inflammatory response by inhibiting the production of other pro-inflammatory cytokines such as IL-1b and TNFα, reducing levels of inflammation and promoting a neuroprotective effect [46].

Considering that IL-6 plays a major role in the development and advancement of schizophrenia [29], it is possible that the behavior of IL-6 levels in response to exercise may explain its beneficial effects on this disease which are evident in many studies. These include improved performance in tasks involving working memory, processing speed, and attentional processes [21], as well as a protective effect on brain plasticity resulting in improvements in the symptomatology of schizophrenia [11].

**Figure 3:**
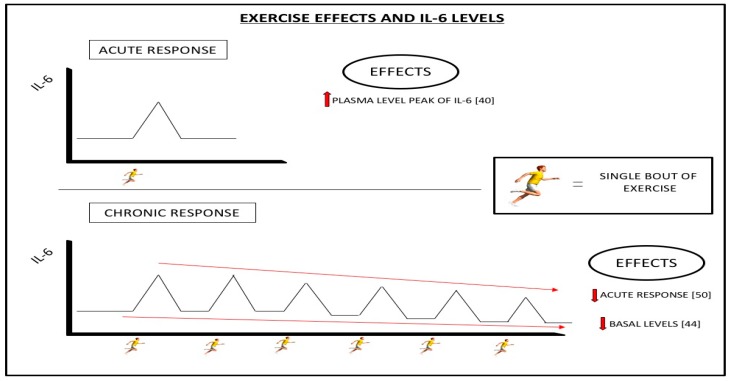
A single bout of exercise causes an increase in plasma levels of IL-6 (ACUTE RESPONSE). However, the regular practice of exercise reduces the acute response to exercise and decreases the basal levels of IL-6 (CHRONIC RESPONSE).

As a stressor, physical exercise would initially cause an acute response leading to an increase in plasma levels of IL-6. Although this may initially be counterproductive in people with schizophrenia (who usually have high IL-6 and inflammation levels), with regular exercise (and the resulting increased adaptative fitness) a chronic response could be achieved. As a result of this chronic response, both the basal IL-6 levels and the peak levels of IL-6 produced during acute responses would decrease in these patients (Figure 3). This double-chronic response would favor both an anti-inflammatory response and a decrease in basal IL-6 levels. This would systemically reduce the state of inflammation which characterizes schizophrenia and favor the exercise-mediated neuroprotective effect on the brain which produces the improvements in cognitive function and symptomatology previously described [11].

Regarding the most appropriate type of exercise, it is still unknown how the different changes in the parameters of the exercise (intensity, duration, density...) specifically affect the levels of IL-6 [13]. Despite this ignorance, recent studies have observed that high-intensity exercise (anaerobic exercise or strength exercise) causes a greater increase in IL-6 levels than low intensity exercise [47], and that eccentric exercise stimulates a greater production of IL-6 than concentric exercise [40].

Currently, most studies use aerobic exercise protocols or concurrent protocols (protocols in which both aerobic and anaerobic exercises are used) [13]. The problem with concurrent protocols is that we cannot know if the effects on IL-6 are due to aerobic exercise or anaerobic exercise.

Because the increase in levels of IL-6 is related to the amount of muscle damage caused by exercise and also because patients with schizophrenia have a state of permanent inflammation, we should stimulate the production of IL-6 progressively with the aim that the consequences of the acute response are not negative.

Therefore, we should start with an aerobic exercise protocol that causes the least possible muscle damage and, consequently, a controlled increase in the levels of IL-6. When patients with schizophrenia adapt to aerobic exercise protocols, we should progressively change to anaerobic exercise protocols (or strength exercise protocols), first with concentric exercise and later with eccentric exercise. With this progression we will achieve that the protocols always provoke a stimulation of IL-6.

## 6. Conclusions

There is now ample evidence for the positive effects that exercise has on schizophrenia. The important role that IL-6 plays in the development and symptomatology of schizophrenia, as well as how exercise can modulate the IL-6 response, is also starting to be understood. Therefore, given the information reviewed here, encouraging the implementation of a regular exercise program with the appropriate training parameters (regarding intensity, duration, type of muscle contractions, etc.) as a treatment for schizophrenia could help to modulate IL-6 levels in these patients, resulting in positive effects by improving both cognitive function and symptomatology owing to cerebral structural changes. Influencing the modulation of IL-6 levels through exercise as a treatment in people with schizophrenia is especially relevant not only because it is an economical and accessible treatment option, but also because standard medication treatments have not so far achieved good results for the negative symptoms of the disease—an outcome which is partially achievable through such exercise programs. More studies will be required to help us understand how the variation in the different exercise parameters affects both the acute and chronic plasma levels of IL-6.
